# Social competition and selection in males and females

**DOI:** 10.1098/rstb.2013.0074

**Published:** 2013-12-05

**Authors:** T. H. Clutton-Brock, E. Huchard

**Affiliations:** Department of Zoology, University of Cambridge, Downing Street, Cambridge CB2 3EJ, UK

**Keywords:** social competition, sexual selection, social selection, mating systems, sex roles, dominance status

## Abstract

During the latter half of the last century, evidence of reproductive competition between males and male selection by females led to the development of a stereotypical view of sex differences that characterized males as competitive and aggressive, and females as passive and choosy, which is currently being revised. Here, we compare social competition and its consequences for selection in males and females and argue that similar selection processes operate in both sexes and that contrasts between the sexes are quantitative rather than qualitative. We suggest that classifications of selection based on distinction between the form of competition or the components of fitness that are involved introduce unnecessary complexities and that the most useful approach in understanding the evolution and distribution of differences and similarities between the sexes is to compare the operation of selection in males and females in different reproductive systems.

## 1. Introduction

Although individuals of both sexes are solitary in some species, members of one or both sexes form temporary or permanent groups in many other species [1,2]. By concentrating individuals in time and space, sociality intensifies competition between them for the resources necessary for survival and reproduction, often increasing the capacity of powerful individuals to obtain a disproportionate share and strengthening selection pressures favouring traits that enhance the success of individuals in competitive encounters [3–5]. While similar processes occur in many social animals, they have been more extensively investigated in mammals than in other animal groups and we draw extensively (but not exclusively) on mammalian examples, though the conclusions that we draw are intended to be general.

Early empirical studies of social competition and its consequences focused principally on males. Their results demonstrated that, by concentrating breeding females, sociality enhanced the ability of individual males to monopolize breeding access to multiple females, favouring the development of polygyny, increasing competition between males for access to female groups and individual females and strengthening selection for male characteristics that confer success in fights or attract potential mating partners [3,6–8]. Comparative studies demonstrated that there were consistent relationships between the size of female groups and the development of secondary sexual characters in males, including increases in relative body size, the size and elaboration of male weaponry (such as male horns and canine teeth) and the extent of male ornaments [9–11].

For a combination of conceptual and practical reasons, few early studies of vertebrates initially explored the causes and consequences of social competition between females. In most groups of animals, the frequency and intensity of aggression is lower in females than in males and secondary sexual characters are generally less developed [12], with the result that their distribution and evolution has attracted less attention than comparable traits in males. In addition, in many polygynous animals, females are harder to recognize individually than males, partly because there are usually large numbers of females within social groups, and partly because they do not exhibit obvious scars as often as males do. Finally, while individual differences in access to reproduction were immediately obvious in males as a result of their differential ability to monopolize groups of females, in species where females breed annually and have relatively long lifespans, the magnitude of individual differences in female fitness is not obvious unless the breeding success of recognizable animals has been monitored over several seasons [13,14]. Most early studies of polygynous vertebrates lacked information of this kind and as a result, did not initially appreciate the extent of individual differences in breeding success among females or their causes.

As field studies developed, the prevalence and intensity of competition between females and the magnitude of individual differences in breeding success among females became clear [4,15–17]. In particular, field studies of social mammals, including rodents [18,19], ungulates [20], carnivores [21,22] and primates [23–26], showed that, where multiple breeding females live in stable groups, reproductive competition between them is often intense and that individual differences in competitive success are often associated with substantial differences in the lifetime reproductive output of females which, in some cases, approach or even exceed those in males [27,28]. It also became clear that, as in males, contrasts in the intensity of competition between females and the development of associated traits reflect variation in social organization and mating systems. Early studies of birds recognized that the evolution of bright plumage in both sexes occurred in species where both females and males were involved in aggressive or territorial displays [17,29] and that greater development of ornamentation in females than males occurs in some species with polyandrous mating systems [7,8,30]. Studies of primate societies further showed that there were consistent relationships between the development of female weaponry and female ornaments, the form and intensity of reproductive competition between females and the structure of social groups [31,32]. The extensive influence of contrasts in social organization and social competition on the evolution of weaponry and ornamentation in both sexes was recognized in important reviews by West-Eberhard [4,17], which emphasized the fundamental similarities in the evolutionary processes operating in males and females and drew attention to parallels between the effects of intrasexual competition and those of competition between juveniles.

More recently, field studies of socially monogamous birds have produced evidence of the importance of competitive displays and mutual mate choice in species where both sexes are ornamented or brightly coloured [33,34]. In addition, studies of cooperative mammals, where young produced by a single breeding female in each group are reared by other group members, have shown that individual differences in breeding success among females are often as large or larger than in males and are associated with intense competition between females for reproductive opportunities [28,35–37]. Finally, research on a variety of animals (including insects, fish and birds) has shown that sex differences in the extent of competition for breeding commonly vary between and within populations in relation to the relative abundance of breeding partners [38–40].

One result of increasing recognition of the extent of social competition between females and its evolutionary consequences has been that the dichotomous characterization of males as competitive and aggressive and females as pacific and choosy has been replaced by the realization that reproductive competition and mate choice (and characteristics associated with them) are widespread in both sexes [41]. Over the past decade, this has increased recognition of the qualitative similarities between the evolutionary processes operating in both sexes and led to a re-evaluation of the operation of sexual selection in males and females [34,42–49]. More recently, it has led to suggestions that it may be useful to distinguish between selection operating through ecological and social competition and to categorize selection pressures according to the different forms of social competition that are involved [4,17,34,45].

In §2, we describe four different forms of social competition that are common in both male and female vertebrates (competitive displays, fighting, competition for social rank and the suppression of reproduction by rivals) and explore their consequences for the evolution of social adaptations and secondary sexual traits in both sexes. Subsequently, we compare the relationship between social competition and selection in the two sexes. Finally, we review the problems associated with attempts to classify different forms of selection and suggest that the most useful approach may be to recognize that natural selection is a single process that operates in diverse ways through multiple components of fitness in both sexes.

## 2. Social competition in males and females

### (a) Displays and ornaments

Competitive male displays are widespread in social animals and are used both to attract breeding partners and to repel rivals [6,50]. Visual, vocal and olfactory displays are often combined and frequently reflect the signaller's hormonal status, condition and physical strength [51–53]. For example, in ring-tailed lemurs, the scent marks of males provide information about the age, condition, status, androgen levels and relatedness of individuals [54,55]. Similarly, in baboons, the loud calls of males reflect their age, rank and physical condition [52,56]. Male displays frequently emphasize male weaponry (including teeth, horns and antlers) as well as male ornaments (including bright or elaborate plumage and pelage) [4,6,34]. While male ornamentation has evolved in some monogamous species, it is more highly developed in species with polygynous breeding systems, and especially in those where multiple males display simultaneously to females [6,57,58]. Especially in seasonal breeders, the frequency of male displays can be high, and in some species, displaying males cease feeding altogether with the result that they are unable to sustain continual reproductive activity for more than a few weeks [57,59].

Like males, females use a combination of visual, vocal and olfactory displays and the frequency and quality of displays signal their age, size and condition [4,17,34,43]. Where both sexes contribute to the defence of breeding territories, female displays are sometimes directed principally at rivals, but in many species, they are also used to attract potential breeding partners and reflect the signaller's fecundity. For example, in some cercopithecine primates, the facial colouration of females is brighter, as in rhesus macaques [60], while in several baboon and macaque species, the structure of copulatory calls given by females changes with the female's stage of oestrus [61,62]. Playback experiments show that males discriminate between calls given by females at different stages of their cycle and are most attracted to the calls of females in late oestrus [62].

Reproductive competition between females breeding in groups can also lead to the development of prominent female ornaments, especially in societies where females compete to attract males and gain direct fitness benefits by breeding with multiple partners [43]. For example, female ornamentation is highly developed in a number of fish and shorebirds where males are primarily responsible for parental care and females compete for access to breeding partners [63,64]. Among socially monogamous birds, female ornamentation appears to be more frequent and more highly developed in species where breeding pairs form groups or colonies than in species where pairs defend separate territories [17,65].

A similar association between group-living and female ornamentation may occur in mammals. One of the most striking examples of female ornamentation are the cyclical perineal swellings found in primate species where females live in groups that include multiple breeding males and commonly mate with multiple partners [31,66]. In these species, females may increase their fitness by attracting and mating with multiple males either because this increases their fecundity directly if they increase their chance of conceiving or obtain better genes for their offspring, or through the social support that they or their offspring receive from males who have mated with them [66–70]. The size of a female's swelling increases as she approaches ovulation [71–74] and large swellings attract males [68,72,73]. In addition, some studies of baboons have found that individual differences in the size of female swellings are correlated with individual differences in fecundity and have suggested that swellings may have originated as a signal of receptivity and subsequently evolved to signal differences in individual quality [68,69]. Whatever the precise mechanism responsible for the evolution of swellings, their elaboration suggests that they are a consequence of competitive signalling that affects the access of females to limited resources that have an important influence on their fitness [69]. Since female cycles are seldom closely synchronized in these species and it seems unlikely that access to sperm limits female reproduction and the most likely explanation is that females compete to attract males in order to increase investment by males in protecting them or their offspring [67].

While both prolonged competitive displays and elaborate ornaments can be highly developed among females, they are not found in all species where reproductive competition between females is intense. For example, neither elaborate visual displays nor elaborate ornaments are obvious in singular cooperative breeders where reproductive skew among females is unusually large, and competition between females is intense [28,36]. However, little is known of the olfactory displays of females in these species and it may be the case that these are highly developed and play a similar role to the visual displays of female primates or birds. Finally, where female ornaments, like the perineal swellings, or copulatory calls produced by female primates signal cyclical changes in fertility, the costs of their production and maintenance often occur at different times from the principal costs of reproduction, with the result that their fitness costs may be low.

### (b) Fighting and weaponry

**Figure 1.**
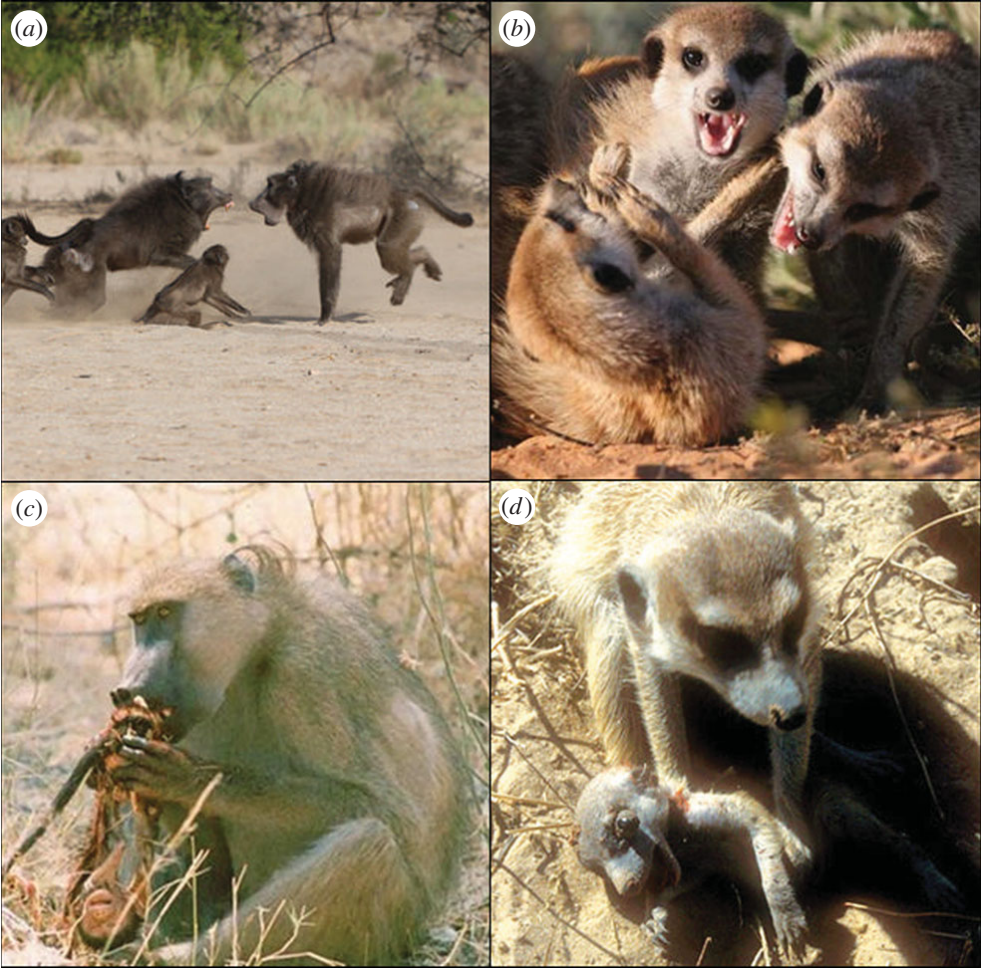
Modes of reproductive competition in males and females. Fights among males are frequent and severe in many polygynous species, as in chacma baboons (*Papio ursinus*, (*a*), picture credit: Elise Huchard) but also occur among females, as in meerkats (*Suricata suricatta*, (*b*), picture credit: Andrew Young). Male infanticide is common in many mammals, including many polygynous primates, such as chacma baboons ((*c*), picture credit: Ryne A. Palombit), whereas female infanticide is also common in other species, including meerkats ((*d*), picture credit: Andrew Young). (Online version in colour.)

Escalated fights between individuals of the same sex occur in both sexes, although their frequency varies (figure 1). Field studies of polygynous species have shown that aggression among males is often frequent and can lead to sustained physical fights, lasting until the defeat of a rival, accompanied by severe, sometimes fatal, wounding [75,76]. Fighting among males typically peaks during periods of reproduction and commonly involves conflicts over access to females, though fights also occur over access to territories, resources and social rank and fighting success affects multiple components of male fitness [13,77]. Where males compete independently, differences in age, size, weight and stamina between contestants commonly play an important role in determining outcomes [75,78]. Fighting and other forms of reproductive competition between males are particularly frequent in seasonal breeders with polygynous mating systems where individual males can monopolize access to multiple females, as in many of the ungulates [59,75] and seals [79].

Fighting is also common between females [43,46,49] (figure 1). In many solitary species, as well as in monogamous ones, females can be as aggressive as males in territorial disputes, and as in males fighting can lead to serious injuries or even death. For example, in owl monkeys, both females and males fight to evict intruders of the same sex, wounding is common and losing can have fatal consequences in both sexes [80]. Similarly, in singular cooperative breeders, like naked mole-rats and meerkats, breeding females are usually intolerant of each other and the death of a dominant breeder is often followed by repeated and protracted fighting between individuals competing for her position [28,37]. Fighting between females is also common in many plural breeders and peaks during the reproductive season [81–83], and here, too, can lead to wounding or death [28,84]. As in males, intraspecific variation in female aggression is often associated with increased levels of testosterone, though this is not so in all species [85–87].

While it is a mistake to characterize females as pacific and competition between them for resources or mating partners is not uncommon [88,89], in most species, escalated fights between females are less frequent and are shorter than fights involving males and serious wounding is not as common [44,76,83]. There are several reasons why physical attacks may be less frequent and less intense in females than males. In some polygynous species, the immediate fitness gains (in the form of extra mating opportunities) that males can achieve as the result of a successful fight may often be greater than the potential benefits of winning fights to females [8,90]. However, where females fight for breeding status and the breeding lifespans of females are longer than those of males, the outcome of fights may have longer lasting effects on breeding success in females than males, and sex differences in the lifetime reproductive benefits of winning fights are likely to be smaller than sex differences in immediate reproductive benefits [28]. In addition, the cumulative costs associated with escalated fights may often be higher for females than for males, as they may incur fatal injuries that affect the survival of dependent offspring: for example, territorial fights among females frequently result in infant deaths in ring-tailed lemurs [91]. Finally, where females are philopatric (as in many mammals), females may be able to control the development or the presence of potential rivals, so that escalated conflicts between individuals of approximately equal physical strength are less common than in males [42].

**Figure 2.**
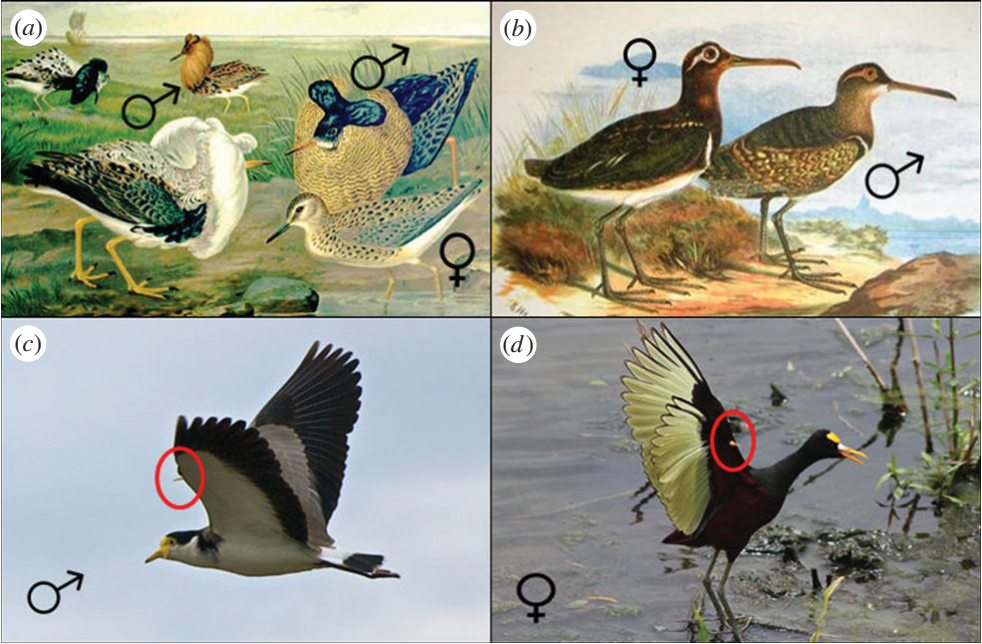
Ornaments and armaments in shorebirds. Males are more highly ornamented in many polygynous shorebirds where they compete with each other on leks in order to gain sexual access to females, as in ruffs (*Philomachus pugnax*, (*a*), illustration of a lek by Johann Friedrich Naumann), whereas females are more highly ornamented than males in some polyandrous species where they compete with each other to gain access to paternal care, as in the painted snipe (*Rostratula benghalensis*, (*b*), illustration by S. Herbert). Similarly, armaments (here wing spurs highlighted by circles) are more developed in males than in females in polygynous species, as in the masked lapwing (*Vanellus miles*, (*c*), picture credit: Gary Stockton/CC BY-NC-ND 2.0) but are more highly developed in females in some polyandrous species, as in the northern jacana (*Jacana spinosa*, (*d*), picture credit: Benjamin Keen/CC-BY-SA-3.0). (Online version in colour.)

The larger benefits of winning fights in males have commonly led to the greater development of secondary sexual characters in males in many polygynous species. In many polygynous and promiscuous mammals, males are larger than females and have more highly developed weapons [9,10,92]. While the relative intensity of social competition between males often exerts an important influence on the evolution of sexual dimorphism, variation in the intensity of social competition between females also plays an important role. As might be expected, sexual dimorphism in body size is often reduced in species where reproductive competition between females is intense and is reversed in some species, including some fish where males are responsible for parental care, several polyandrous shorebirds and a number of social mammals [6,42,44,93]. There is also evidence that increased reproductive competition between females can be associated with increased development of weaponry in females. For example, in monogamous primates where females defend feeding territories against neighbours, females have (relatively) larger canines than in species where females seldom contribute to territorial defence [32,94]. Similarly, in some polyandrous shorebirds where males are responsible for parental care and females compete intensely for breeding partners, females show greater development of wing spurs used in intrasexual fights [95] (figure 2).

Although there are examples where weaponry is more highly developed in females than in males, these are rare, even among species where reproductive competition is more intense among females than among males (see review by Young & Bennett [96]). The lower frequency and duration of fights between females (see above) may help to explain why this is the case. In addition, in some cases, the costs of developing or maintaining weaponry may be greater in females than in males. Alternatively, the form of intrasexual fights can differ between the sexes, favouring the development of different characteristics in males and females [46,49] or selection for the development of weapons in females may be reinforced by their use in the defence of offspring against predators or conspecifics [97]. Finally, intense intrasexual competition between females may sometimes favour sexual mimicry rather than dimorphism. For example, in some plural breeders where female competition is intense, females show heightened testosterone levels at particular stages of the breeding cycle [86,98] and their genitalia show signs of masculinization [99–102]. Though masculinization of female genitalia may sometimes be a non-adaptive by-product of elevated testosterone levels or of increased sensitivity to androgens [103,104], sexual mimicry may also allow females to deflect aggression directed at them by dominant females or males or to control the identity of mating partners [105–107].

### (c) Dominance and reproductive success

Where individuals live in stable groups, they are often able to identify each other and avoid escalated fights with individuals that have recently beaten them, so that dominance hierarchies develop [31,108,109]. Many early studies of vertebrates (and of mammals in particular) focused on species where breeding groups included multiple breeding males, including studies of wild sheep [108], deer [110], baboons [111,112], gorillas [113] and chimpanzees [114,115] and documented regular dominance relationships among males. In some species, male dominance and success in fights are effectively inseparable: for example, in red deer, bighorn sheep, elephants and many social primates, the social rank of males depends on their fighting ability, and losers suffer an immediate change in status relative to the winner and may be evicted from breeding groups [59,116–120].

Where male dominance rank depends on fighting success, the rank of males is often associated with their age, size and weight [78,79,116,121]. For example, in sexually dimorphic ungulates, where fights between males involve pushing or ramming contests, the outcome of fights often depends on the relative size and weight of contestants [27,59,118]. However, male size is not always important and its influence on dominance status depends on fighting techniques. For example, in horses, where individuals fight by biting their rivals, dominance is unrelated to body size [122]. Similarly, in social primates where males form coalitions to compete for status or access to females, the rank and social connections of allies have a more important influence on the rank and breeding success of males than their body size or condition [123–125]. For example, in Assamese macaques, the breeding success of males that have dispersing from their natal group is correlated with the strength and number of their social bonds with other males [126].

Male dominance rank is often positively correlated with access to receptive females and with mating frequency [112,116,127–130], though the strength of correlations between rank and mating success and the extent of reproductive skew among males varies widely as a result of female reproductive synchrony and female mating preferences [116,130–132]. In addition, high rank often affects access to resources as well as to alliance partners [117,121,133] and is frequently associated with benefits to health and survival [134–136]. However, high-ranking males are commonly involved in more frequent exchanges of aggression than low-ranking individuals and are more likely to be wounded [76,83] so that there are likely to be trade-offs between the relative status of males and the period for which they maintain their rank [116].

Dominance hierarchies are also common among females, though they do not occur in all species and the frequency, regularity of outcome and linearity of hierarchies vary widely between and within species [42,137,138]. As in males, rank is often established through physical contests [28,42,139] and is frequently associated with age, body size or mass [140–142]. In plural breeders where females are philopatric, adult females frequently support their daughters and other members of their group and these interventions help to establish the eventual rank and breeding success of juveniles [21,143–145]. Although females commonly support close matrilineal relatives [146–148], they can also form social bonds with unrelated individuals which may also affect their social rank [23,24,149–151].

As in males, dominance rank in females is usually positively correlated with reproductive success as well as with access to resources, though relationships vary widely in strength and have not been found in all studies [42,49,129,130,152,153]. For example, in several cercopithecine primates, high-ranking females breed earlier and more frequently, their offspring grow faster and are more likely to survive and breed successfully than those of subordinate females [153–161]. Similarly in spotted hyenas, high-ranking females have priority of access at kills, breed at younger ages, wean their offspring more rapidly, breed more frequently and produce more surviving offspring than subordinate females [21,162,163]. In some social primates, high-ranking females and their offspring are also less likely to be evicted from social groups [153], and like dominant males, show improved health and survival [135,136].

While the determinants and consequences of rank are similar in the two sexes, the strength of relationships between fighting ability, rank and reproductive success often appears to differ. Although direct comparisons of the effects of rank on fitness in males and females are scarce, the effects of fighting ability and physical strength often appear to be stronger in males than in females, where rank often depends to a greater extent on social bonds and coalitionary support [25,148–150,164,165]. Rank dependency on social support may be particularly pronounced among members of the philopatric sex but can also occur in the dispersing sex [122,126]. Dominance rank in females tends to be more stable than among males: female rank is often established early in life and persists through old age [166,167], whereas the rank of males commonly changes throughout their life and the period over which individuals hold high ranks is often short [112,116,168]. The contrast in the stability of rank in the two sexes may be related to the stronger effects of kin support in females as well as to the greater intensity of reproductive competition and the greater costs of maintaining high social status in males [28].

Since no direct comparisons of the effects of social rank on the reproductive success of individuals of both sexes and on the fitness of their progeny are yet available, it is not yet possible to come to any firm conclusion concerning the relative intensity of selection on traits associated with social rank in the two sexes. It is commonly suggested that the reproductive benefits of dominance are greater in males than in females [129,130], and this may generally be the case, with the possible exception of polyandrous species and singular, cooperative breeders [35]. However, recent studies suggest that sex differences in the effects of social status on fitness are likely to vary and may often be smaller than has generally been assumed as a result of sex differences in the duration of breeding lifespans [42,49].

### (d) Reproductive suppression

**Figure 3.**
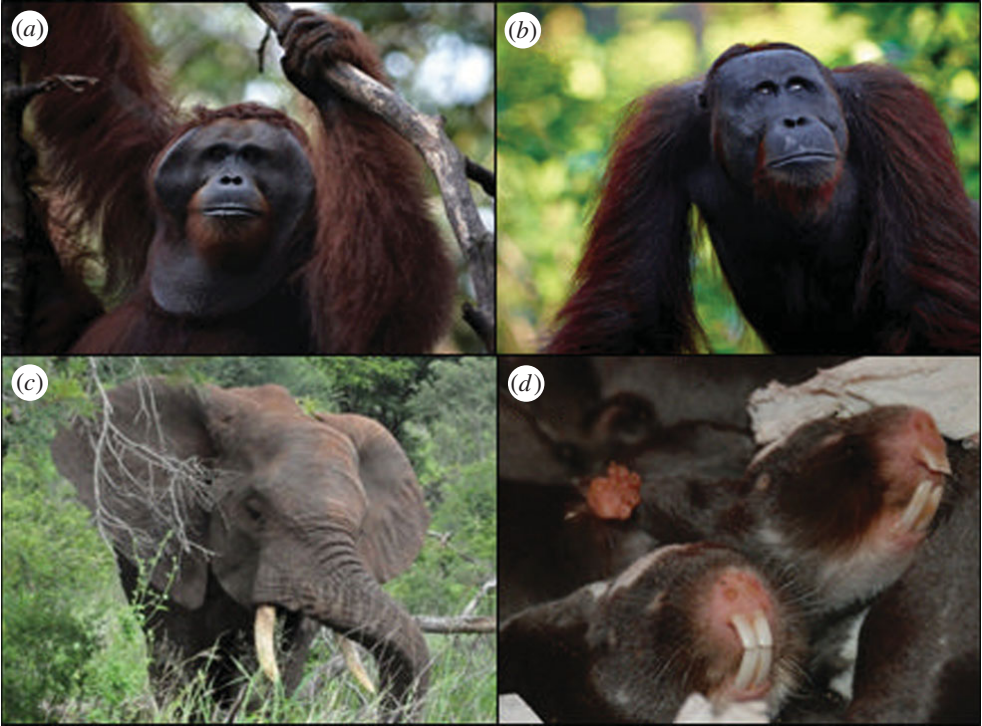
Physiological suppression of reproductive function in males and females. The physiological suppression of reproductive function is common among males, even in species where males are solitary, as in orangutans (*Pongo pygmaeus*), where large males with pronounced secondary sexual characters ((*a*), picture credit: Michael Malherbe) suppress the development and reproductive function of younger males ((*b*), picture credit: Michael Malherbe) living in overlapping homeranges or in elephants (*Loxodonta africana*, (*c*), picture credit: Elise Huchard). Reproductive suppression is also common among females in singular breeders like Damaraland mole-rats (*Fukomys damarensis*, (*d*), picture credit: Markus Zoettl). (Online version in colour.)

In addition to enhancing their social rank and reproductive success by winning physical contests and rising in the social hierarchy, both males and females can also enhance their own reproductive success by evicting rivals or suppressing their reproductive attempts (figure 3). In many species, adult males also evict adolescents of the same sex [169–173] who often show heightened mortality levels before they are integrated into a new breeding group [174]. Where dominant males tolerate the presence of younger individuals, aggression directed at younger males, or in some cases, the presence of older and more dominant males, can affect the hormonal status of younger males and retard or depress their sexual behaviour. For example, in African elephants, interactions with older dominant males can ‘switch off’ reproductive activity (‘musth’) in younger males [119,175]. Interactions between males can also delay the development of subordinates, with the result that subordinates often show reduced body mass, condition and gonad size, less active scent glands, reduced development of secondary sexual traits, decreased levels of reproductive and growth hormones and lower frequencies of sexual behaviour [176–179]. Eviction or reproductive suppression among males is particularly prevalent in systems where dominant males cannot guard receptive females effectively or where scramble competition for breeding opportunities is important. In addition, reproductive suppression may benefit dominant males by ensuring that subordinate males are less attractive to females [180] or suffer disadvantages in sperm competition [181] as well as by reducing the costs of maintaining dominance and of mate-guarding to alpha males.

In some mammalian species, males kill unrelated dependent juveniles and this provides them with additional mating opportunities by allowing them to impregnate the victim's mother, who typically resumes to oestrus within a few weeks after losing a dependent offspring [182,183]. Infanticide often involves males that have recently immigrated into social groups, but also occurs in societies where several males are associated with groups of females [184]. Male infanticide is most frequent where male tenure is short, females have a long lactational infertility and infanticidal males can gain sexual access to the mother of the killed infant [185,186]. By contrast, it is unusual where females conceive immediately after giving birth (as in equids, some terrestrial carnivores and many seals). Some observations suggest that males may preferentially target future rivals: for example, males appear to kill male offspring more often than female offspring in chimpanzees but the generality of this trend and the reasons for it are not yet clear [187,188].

While adult males are frequently intolerant of each other's presence, they can be more tolerant of the presence of kin than non-kin. For example, in multi-male coteries of prairie dogs, relationships between resident males are more amicable and less competitive when males are close kin than when they are unrelated [78]. Similarly, in alpine marmots, dominant males are more aggressive with unrelated subordinates, and suppress their development to a greater extent than that of related subordinates [189]. In chimpanzees, too, maternally related males are more likely to affiliate with and support each other than the offspring of unrelated females, although not all allies are related [190]. However, kinship appears to affect some forms of cooperation more than others. For example, while male chimpanzees selectively support related males in competitive encounters with other males, there is no evidence of kin-bias in hunting behaviour and males are no more likely to share meat with maternal kin than with unrelated males [191].

Females adopt many of the same tactics to suppress development and reproduction by rivals. In singular breeders, dominant females commonly evict subordinates from the group when they reach adolescence, approach adult size or attempt to breed [28,192]. Suppression of reproductive function in subordinates is at least as common among females as among males. In many singular breeders, dominant females direct regular aggression at older adolescents which can reduce levels of reproductive hormones and delay their development [179]. In some plural breeders, too, dominant females direct frequent aggression at subordinates which delays their development, disrupts their reproductive cycles and causes them to down-regulate their reproductive systems or abort litters [179,193]. For example, in yellow baboons, dominant females direct frequent aggression at subordinate females during the follicular phase of their cycles, raising the number of cycles before they conceive [26]. Aggression directed at females shortly after mating can reduce implantation success and induce abortion [42,194,195].

Like males, females are often more tolerant of individuals of the same sex if they are close relatives than if they are more distant relatives or unrelated. In some voles, females preferentially settle close to relatives and those with ranges abutting those of relatives breed at younger ages, rear more offspring and have higher rates of survival to the next breeding season than those with ranges abutting those of unrelated individuals [196,197]. Similarly, in meerkats, the probability that a dominant female will evict a subordinate increase as her coefficient of relatedness to the dominant female falls [198]. In some cases, the suppression of subordinate development and reproduction eases where group size is low or food availability is high, suggesting that dominant females adjust their behaviour to the availability of resources [35,198].

Infanticide by females is also widespread in many social species and is probably more frequent than infanticide by males [199,200]. For example, in black-tailed prairie dogs, females commonly kill the offspring of subordinates occupying neighbouring burrows, even if they are close relatives [78]. Like males, females may preferentially target their future rivals. For example, in some cercopithecine monkeys, where females are philopatric and the relative rank and reproductive success of individuals depends on the rank and size of their matrilineal group, mothers direct higher rates of aggression at the daughters of subordinate females than at their sons [201], generating higher frequencies of mortality in female offspring [202,203]. While female infanticide has been extensively documented in mammals, it also occurs in other groups. For example, in some polyandrous birds, females who acquire breeding territories destroy nests or kill the dependent young of their rival and their behaviour parallels that of males in polygynous mammals [204,205].

While individuals of both sexes commonly evict rivals, sex differences in the frequency of eviction and in the intensity of overt competition between group members often appear to be greatest in whichever sex most frequently remains and breeds in their natal group. For example, in many social animals where females typically remain and breed in their natal group while males disperse, protracted conflicts over group membership are more frequent between females than between males while the reverse may be usual where males are commonly philopatric [35,206,207]. This may be partly because members of the dispersing sex are unlikely to invest heavily in competition to remain in the group, or established adults are less likely to react aggressively to natals of the dispersing sex which do not represent serious competitors because they are closely related to residents of the opposite sex who are unlikely to mate with them. In addition, increased conflict may occur between individuals of the philopatric sex because, if they do leave their own group, they are frequently prevented from joining established breeding groups and are consequently less willing to leave [206,208].

## 3. Social competition and selection in males and females

This brief survey emphasizes the importance and similarity of social competition in both sexes as well as its pervasive impact on selection pressures. In group-living animals, competition for resources of all kinds is mediated by social mechanisms operating within the group they live in. Although competition between males for mates and breeding territories is common, females, too, frequently compete intensely for breeding sites, reproductive opportunities, membership of breeding groups or social status within them. Social competition also occurs between groups. In many animals, members of different breeding groups compete with each other for resources or space and frequently interfere with each other's breeding attempts. Competition between groups as well as between residents and intruders exerts an important influence on the evolution of group size and dispersal patterns as a result of the pervasive tendency for larger social units to displace smaller ones [209,210]. Similarly, where both sexes are solitary, social interactions between neighbours or between residents and intruders exert an important influence on the reproductive success of individuals.

Although studies of competition most commonly focus on adults, social competition can occur at all stages of development [4,17]. In some mammals, competition between litter-mates begins before birth and persists throughout the period of lactation and early development, affecting both the survival of neonates and juveniles and their subsequent reproductive success and longevity [162,202] and in some cases, it can lead to unusual patterns of development, such as the precocious development of teeth in spotted hyenas associated with intense sibling rivalry [211] or the striking natal coats of infants in some primates [4,17,212]. In others, social competition can lead to the development of traits that mimic the characteristics of the opposite sex, as in fossas and in spotted hyenas [42,107].

The common property in all these cases is that competing individuals belonging to the same subdivision of the population (which may be a deme, a group, a cohort or a litter) play repeated zero-sum games with each other, which involve fights over high-value resources that are seldom shared between competitors, and some individuals consistently win these contests, generating large individual differences in fitness. Where individuals engage simultaneously or successively in competitive interactions with many competitors and can monopolize a large proportion of available resources or breeding opportunities, competition is likely to be particularly intense and selection for traits that increase competitive ability is likely to be extremely strong. The effects of competitive success are seldom limited to a single component of fitness and commonly influence the growth, fecundity and survival of individuals as well as the fitness of their offspring. In addition, social competition often mediates the effects on fitness components of a wide range of environmental challenges, including starvation, disease [135,213,214] and predation [215,216]. As a result, in group-living species, it is often difficult to conceive of selection pressures that are unaffected by social competition with the result that the distinction between ‘social’ and ‘ecological’ selection proposed by West-Eberhard [4,17] and others [34,45] is impractical.

As the previous sections show, recent research emphasizes the fundamental similarity in the causes of social competition in the two sexes and emphasizes that most contrasts between the sexes are quantitative rather than qualitative, matters of degree rather than differences in kind. In both sexes, the intensity of reproductive competition is determined partly by contrasts in the number of individuals competing for breeding partners as a result of variation in the Operational Sex Ratio, generated by sex differences in the time required by individuals to recover from an attempt to reproduce (‘time out’ or ‘dry time’) [39,217,218] and by variation in the number of individuals whose sexual development is delayed or suppressed; partly by relationships between competitive success and reproductive success in the two sexes, including Bateman gradients [8,40,218]; and partly by factors affecting the ability of individuals to monopolize breeding partners and resources necessary for reproduction [7,43,210,219].

The extent of sex differences in behaviour and competition and in the selection pressures these generate is in the process of being re-valuated in the light of more precise and more extensive data from natural populations. For example, while it has been widely accepted that investment in competitive displays, in fighting and in competition for dominance status is often greater in males than in females, aggressive competition is also frequent among females and can be more intense than among males [46,49]. We still know relatively little about the effects of variation in competitive success on fitness in the two sexes or about trade-offs between the capacity of individuals to monopolize breeding opportunities at particular stages of the lifespan and the duration of effective breeding, which often contributes a substantial proportion of variation in lifetime reproductive success [16,116]. For example, no study of a social vertebrate has yet been able to make a direct comparison of relative effects of social rank on peak breeding success, on the duration of successful breeding and on lifetime reproductive success in both sexes.

The consistency of sex differences in social competition is also being re-evaluated. Several recent studies have shown that sex differences in reproductive competition differ between populations as well as within the same population over time [38,40,43,116] but we know little of the extent or the distribution of these differences. Similarly, while it is widely accepted that mating preferences are usually more highly developed in females than males and individual variation in attractiveness is commonly a more important cause of variation in fitness in males than in females [6,220], there is increasing evidence that the strength of female mating preferences varies between and within species [221,222] and that males, too, often show consistent mating preferences [223,224] that can lead to the evolution of conspicuous ornaments in females [33,65,66,225].

Finally, sex differences in the extent of individual differences in breeding success and in the potential strength of selection pressures are being re-assessed. While it is generally assumed that in polygynous and promiscuous species individual differences in the potential pay-offs of successful competition are larger in males than females, the shorter breeding lifespans of males in these species combined with the consistency of individual differences in breeding success among females mean that sex differences in the extent to which lifetime breeding success differs between individuals may often be relatively small [16,49] and can be reversed in species where alloparental care alleviates the costs of maternal investment [28,35,36]. Moreover, transgenerational maternal effects on offspring phenotypes [226–228] may result in a greater covariance between maternal and offspring fitness than between paternal and offspring fitness and further attenuate contrasts in variance in reproductive success between the sexes after several generations [49].

## 4. Categories of selection

The underlying similarity in the operation of selection in males and females has recently generated discussion of the distinction between natural selection and sexual selection [34,43–46,48,49,229–232]. Darwin developed the theory of sexual selection to account for the evolution of secondary sexual characters and was well aware that they occur in both sexes. Although the *Descent of Man* focuses primarily on the evolution of secondary sexual traits in males as a result of mating competition, he appreciated that both of the two forms of sexual selection that he described (intrasexual competition to breed and intersexual mate choice) can occur in both sexes and can lead to the evolution of secondary sexual characters. For example, in the General Summary of the *Descent of Man*, he describes the operation of sexual selection in more general terms than he uses in chapters where he focuses on the evolution of secondary sexual characters in males: ‘*Sexual selection depends on the success of certain individuals over others of the same sex, in relation to the propagation of the species. The sexual struggle is of two kinds; in the one it is between the individuals of the same sex, generally the males, in order to drive away or kill their rivals, the females remaining passive; whilst, in the other, the struggle is likewise between the individuals of the same sex, generally the females, which no longer remain passive, but select the more agreeable partners*’ (our italics). However, in line with Darwin's more specific descriptions, sexual selection is now usually defined as operating exclusively through intrasexual competition for mating opportunities or through competition for access to gametes of the opposite sex [6,48,218].

While definitions of sexual selection that restrict it to selection operating through mating success offer greater precision, they have the disadvantage that they limit the effective role of sexual selection in females, where direct competition is usually over opportunities to breed or resources necessary for reproduction (including paternal investment) rather than over access to males or to sperm [46,49]. As a result, most examples of selection operating through social competition between females, including selection favouring female ornaments or weapons that allow individuals to compete successfully for nuptial gifts or male investment in offspring [233,234], would be excluded and the evolution of traits serving similar functions in males and females would have to be attributed to different evolutionary processes.

**Figure 4.**
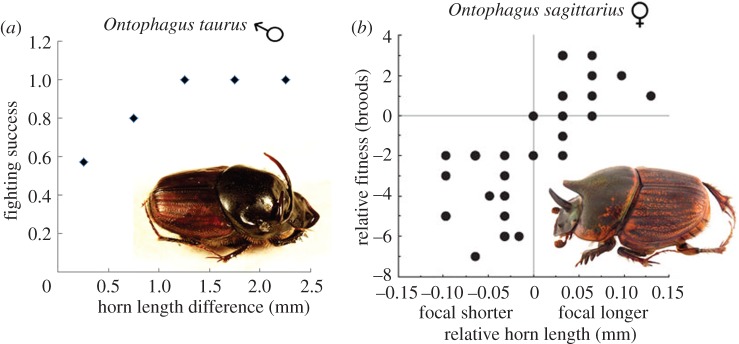
Horn length and fighting success in male and female *Ontophagus* beetles. (*a*) Relationship between male fighting success and horn length in *Ontophagus taurus* (*n* = 27 contests). Graph adapted from Moczek & Emlen [235], fig. 3. Picture credit: Tom Murray. (*b*) Relationship between relative horn length and relative fitness of competing females in *O. sagittarius*. Positive values on the *x*- and *y*-axes represent cases where, in pairs of competing females matched for body size, the focal female had a larger horn and produced more broods relative to her competitor. Graph reproduced from Watson & Simmons [236], fig. 3. Picture credit: Udo Smidt. (Online version in colour.)

Perhaps the example that crystallizes this dilemma most clearly is a study of *Onthophagus* dung beetles where both sexes have evolved large horns that are used in contests over access to dung that is used to form balls where eggs are laid (figure 4). Competition for dung leads to selection operating through individual differences in mating success in males and through individual differences in fecundity in females, and for this reason, advocates of a narrow definition of sexual selection regard horns in males as a product of sexual selection but attribute their evolution in females to other evolutionary processes, such as social selection [34]. While it is certainly possible to draw this distinction, it is difficult to avoid thinking that future generations of evolutionary biologists will be puzzled to find that similar traits in males and females, generated by identical forms of competition for the same resource are attributed to different evolutionary processes.

One possible solution to the semantic problem raised by these comparisons is to use sexual selection to refer to all selection pressures that are influenced by the sex of individuals [49,229,237]. However, this has the disadvantage that, in sexual organisms, few (if any) selection pressures are unaffected by the sex of individuals. Alternatively, sexual selection might be used either to refer to all selection pressures operating through intrasexual competition to breed (rather than to mate) or to all selection pressures favouring the evolution of secondary sexual characters [43,44]. This, however, would include selection operating through female competition for resources and would introduce the difficulty of distinguishing reproductive competition from competition for resources necessary for survival [43,48].

Recently, two reviews have advocated using the framework proposed by West-Eberhard in 1983, involving an initial division of selection pressures into social selection (those operating through all forms of social competition) and selection pressures operating through other forms of competition, as well as a secondary division of social selection into selection pressures operating through competition for mating opportunities (sexual social selection) and those operating through all other forms of social competition (non-sexual social selection) [34,45]. For enthusiasts, they also offer the possibility of further subdivisions, including mutual sexual selection, mutual social selection, individual social selection and indirect social selection [34].

While it is useful to recognize the diversity of ways in which social competition can operate, this approach, too, introduces unnecessary complexities and has substantial disadvantages [230]. In group-living species, social competition affects all selection pressures so that it is unclear what social selection excludes. Moreover, as successful social competition commonly affects multiple components of fitness in both sexes, there are practical difficulties in attributing the evolutionary processes responsible for particular traits to sexual social selection versus non-sexual social selection: for example, should male traits that increase competitive success (like large body size or large canines) be regarded as products of sexual social selection (because they affect mating success) or as products of non-sexual social selection (because they affect survival)? Finally, even if it was possible to distinguish clearly between sexual social selection and non-sexual social selection, similar adaptations to reproductive competition in males and females (like the horns of *Onthophagus* beetles) would still be attributed to different evolutionary processes, extending an unnecessary and stereotypical distinction between the evolutionary processes operating in the two sexes.

The difficulties of attempting to distinguish between ecological, social and sexual selection should raise questions about the need to categorize different forms of natural selection and the desirability of doing so. It is now widely recognized that sexual selection is a sub-category of natural selection rather than an alternative process. Recent studies of sexual organisms have shown how selection operates on males and females through many components of fitness at many stages of their lifespans; how it can be driven by competition with different types of competitors for different resources at different stages of the breeding cycle; how its operation varies in strength, direction and consistency and how it can have many different outcomes and consequences. While it can be useful to identify different forms of competition and contrasting types of selection, natural selection is a single process that operates in diverse ways [238]. Contrasts between different types or forms of natural selection are artificial and even the most elaborate classifications of selection pressures are likely to obscure important differences and disguise important similarities, as the recent history of research on sexual selection shows. There is little evidence that selection operating through different forms of social competition has qualitatively different outcomes so, to extend our understanding of the evolution of differences between the sexes, it may be more useful to explore and compare the ways in which natural selection operates in males and females in contrasting systems than to prolong attempts to develop classifications of selection based on the form of competition or the components of fitness that are involved.

## References

[1] Wilson EO 1971 The insect societies. Cambridge, MA: Belknap Press

[2] Wilson EO 1975 Sociobiology. Cambridge, MA: Harvard University Press

[3] Crook JH 1972 Sexual selection, dimorphism and social organization in the primates. In Sexual selection and the descent of man 1871–1971 (ed. Campbell B), pp. 231–281 Chicago, IL: Aldine

[4] West-Eberhard MJ 1979 Sexual selection, social competition and evolution. Proc. R. Soc. Lond. B 123, 222–234

[5] Wynne-Edwards VC 1962 Animal dispersion in relation to social behaviour. Edinburgh, UK: Oliver and Boyd

[6] Andersson M 1994 Sexual selection. Monographs in Behavior and Ecology Princeton, NJ: Princeton University Press

[7] Emlen ST Oring LW 1977 Ecology, sexual selection, and the evolution of mating systems. Science 197, 215–223 (doi:10.1126/science.327542) 327542 10.1126/science.327542

[8] Trivers RL 1972 Parental investment and sexual selection. In Sexual selection and the descent of man, 1871–1971 (ed. Campbell B), pp. 136–179 Chicago, IL: Aldine-Atherton

[9] Alexander RD Hoogland JL Howard RD Noonan KM Sherman PW 1979 Sexual dimorphisms and breeding systems in pinnipeds, ungulates, primates and humans. In Evolutionary biology and human social behavior: an anthropological perspective (eds Chapman NA Irons W), pp. 402–435 North Scituate, MA: Duxbury Press

[10] Clutton-Brock TH Harvey PH Rudder B 1977 Sexual dimorphism, socionomic sex ratio and body weight in primates. Nature 269, 797–800 (doi:10.1038/269797a0) 927503 10.1038/269797a0

[11] Harvey PH Kavanagh M Clutton-Brock TH 1978 Sexual dimorphism in primate teeth. J. Zool. 186, 475–485 (doi:10.1111/j.1469-7998.1978.tb03934.x)

[12] Darwin C 1871 The descent of man, and selection in relation to sex. London, UK: John Murray

[13] Clutton-Brock TH 1988 Reproductive success. Chicago, IL: University of Chicago Press

[14] Clutton-Brock TH 2012 Long-term, individual-based field studies. In Long-term field studies of primates (eds Kappeler PM Watts DP), pp. 437–449 Berlin, Germany: Springer

[15] Clutton-Brock T Sheldon BC 2010 Individuals and populations: the role of long-term, individual-based studies in ecology and evolutionary biology. Trends Ecol. Evol. 25, 562–573 (doi:10.1016/j.tree.2010.08.002) 20828863 10.1016/j.tree.2010.08.002

[16] Clutton-Brock TH 1988 Reproductive success. In Reproductive success (ed. Clutton-Brock TH), pp. 472–486 Chicago, IL: University of Chicago Press

[17] West-Eberhard MJ 1983 Sexual selection, social competition and speciation. Q. Rev. Biol. 55, 155–183 (doi:10.1086/413215)

[18] Hoogland JL 1979 Aggression, ectoparsitism, and other possible costs of prairie dog (Sciuridae: *Cynomys* spp.) coloniality. Behaviour 69, 1–35 (doi:10.1163/156853979X00377)

[19] Sherman PW 1981 Reproductive competition and infanticide in Belding's ground squirrels and other animals. In Natural selection and social behavior (eds Alexander RW Tinkle DW), pp. 311–351 New York, NY: Chivon Press

[20] Clutton-Brock TH 1983 Selection in relation to sex. In Evolution from molecules to men (ed. Bendall BJ), pp. 457–481 Cambridge, UK: Cambridge University Press

[21] Holekamp KE Smale L Szykman M 1996 Rank and reproduction in the female spotted hyaena. J. Reprod. Fert. 108, 229–237 (doi:10.1530/jrf.0.1080229) 10.1530/jrf.0.1080229 9038781

[22] Kruuk H 1972 The spotted hyena: a study of predation and social behaviour. Chicago, IL: University of Chicago Press

[23] Dittus WJP 1979 The evolution of behavior regulating density and age-specific sex ratios in a primate population. Behaviour 69, 265–301 (doi:10.1163/156853979X00511)

[24] Dittus WPJ 1977 The social regulation of population density and age-sex distribution in the toque monkey. Behaviour 63, 281–322 (doi:10.1163/156853977X00450)

[25] Silk JB 1993 The evolution of social conflict among female primates. In Primate social conflict (eds Mason WA Mendoza SP), pp. 49–83 Albany, NJ: State University of New York Press

[26] Wasser SK Starling AK 1988 Proximate and ultimate causes of reproductive suppression among female yellow baboons at Mikumi National Park, Tanzania. Am. J. Primatol. 16, 97–121 (doi:10.1002/ajp.1350160202) 10.1002/ajp.1350160202 31968869

[27] Clutton-Brock TH Albon SD Guinness FE 1988 Reproductive success in male and female red deer. In Reproductive success (ed. Clutton-Brock TH), pp. 325–343 Chicago, IL: University of Chicago Press

[28] Clutton-Brock TH Hodge SJ Spong G Russell AF Jordan NR Bennett NC Sharpe LL Manser MB 2006 Intrasexual competition and sexual selection in cooperative mammals. Nature 444, 1065–1068 (doi:10.1038/nature05386) 17183322 10.1038/nature05386

[29] Huxley JS 1938 Darwin's theory of sexual selection and the data subsumed by it, in the light of recent research. Am. Nat. 72, 416–433 (doi:10.1086/280795)

[30] Jenni DA 1974 Evolution of polyandry in birds. Am. Zool. 14, 129–146

[31] Clutton-Brock TH Harvey PH 1976 Evolutionary rules and primate societies. In Growing points in ethology (eds Bateson PPG Hinde RA), pp. 195–237 Cambridge, UK: Cambridge University Press

[32] Harvey PH Kavanagh M Clutton-Brock TH 1978 Canine tooth size in female primates. Nature 276, 817–818 (doi:10.1038/276817a0) 723954 10.1038/276817a0

[33] Kraaijeveld K Kraaijeveld-Smit FJL Komdeur J 2007 The evolution of mutual ornamentation. Anim. Behav. 74, 657–677 (doi:10.1016/j.anbehav.2006.12.027)

[34] Tobias JA Montgomerie R Lyon BE 2012 The evolution of female ornaments and weaponry: social selection, sexual selection and ecological competition. Phil. Trans. R. Soc. B 367, 2274–2293 (doi:10.1098/rstb.2011.0280) 22777016 10.1098/rstb.2011.0280 PMC3391421

[35] Clutton-Brock T 2009 Structure and function in mammalian societies. Phil. Trans. R. Soc. B 364, 3229–3242 (doi:10.1098/rstb.2009.0120) 19805430 10.1098/rstb.2009.0120 PMC2781877

[36] Hauber ME Lacey EA 2005 Bateman's principle in cooperatively breeding vertebrates: the effects of non-breeding alloparents on variability in female and male reproductive success. Integr. Comp. Biol. 45, 903–914 (doi:10.1093/icb/45.5.903) 21676841 10.1093/icb/45.5.903

[37] Reeve HK Sherman PW 1991 Intracolonial aggression and nepotism by the breeding female naked mole-rat. In The ecology of the naked mole-rat (eds Sherman PW Jarvis JUM Alexander RD), pp. 337–358 Princeton, NJ: Princeton University Press

[38] Forsgren E Amundsen T Borg AA Bjelvenmark J 2004 Unusually dynamic sex roles in a fish. Nature 429, 551–554 (doi:10.1038/nature02562) 15175750 10.1038/nature02562

[39] Kvarnemo C Ahnesjo I 1996 The dynamics of operational sex ratios and competition for mates. Trends Ecol. Evol. 11, 404–408 (doi:10.1016/0169-5347(96)10056-2) 21237898 10.1016/0169-5347(96)10056-2

[40] Simmons LW 1992 Qualification of role reversal in relative parental investment in a bush cricket. Nature 358, 61–63 (doi:10.1038/358061a0)

[41] Karlsson Green K Majidian JA 2011 Active males, reactive females: stereotypic sex roles in sexual conflict research? Anim. Behav. 81, 901–907 (doi:10.1016/j.anbehav.2011.01.033)

[42] Clutton-Brock T Huchard E 2013 Social competition and its consequences in female mammals. J. Zool. 289, 151–171 (doi:10.1111/jzo.12023)

[43] Clutton-Brock TH 2007 Sexual selection in males and females. Science 318, 1882–1885 (doi:10.1126/science.1133311) 18096798 10.1126/science.1133311

[44] Clutton-Brock TH 2009 Sexual selection in females. Anim. Behav. 77, 3–11 (doi:10.1016/j.anbehav.2008.08.026)

[45] Lyon BE Montgomerie R 2012 Sexual selection is a form of social selection. Phil. Trans. R. Soc. B 367, 2266–2273 (doi:10.1098/rstb.2012.0012) 22777015 10.1098/rstb.2012.0012 PMC3391428

[46] Rosvall KA 2011 Intrasexual competition in females: evidence for sexual selection? Behav. Ecol. 22, 1131–1140 (doi:10.1093/beheco/arr106) 22479137 10.1093/beheco/arr106 PMC3199163

[47] Rubenstein DR 2012 Sexual and social competition: broadening perspectives by defining female roles. Phil. Trans. R. Soc. B 367, 2248–2252 (doi:10.1098/rstb.2011.0278) 22777013 10.1098/rstb.2011.0278 PMC3391420

[48] Shuker DM 2010 Sexual selection: endless forms or tangled bank? Anim. Behav. 79, e11–e17 (doi:10.1016/j.anbehav.2009.10.031)

[49] Stockley P Bro-Jorgensen J 2011 Female competition and its evolutionary consequences in mammals. Biol. Rev. 86, 341–366 (doi:10.1111/j.1469-185X.2010.00149.x) 20636474 10.1111/j.1469-185X.2010.00149.x

[50] Bradbury JW Vehrencamp SL 2011 Principles of animal communication. Sunderland, MA: Sinauer Associates

[51] Clutton-Brock TH Albon SD 1979 The roaring of red deer and the evolution of honest advertisement. Behaviour 69, 145–170 (doi:10.1163/156853979X00449)

[52] Fischer J Kitchen DM Seyfarth RM Cheney DL 2004 Baboon loud calls advertise male quality: acoustic features and their relation to rank, age, and exhaustion. Behav. Ecol. Sociobiol. 56, 140–148 (doi:10.1007/s00265-003-0739-4)

[53] Gosling LM Roberts SC 2001 Scent-marking by male mammals: cheat-proof signals to competitors and mates. Adv. Stud. Behav. 30, 169–217 (doi:10.1016/S0065-3454(01)80007-3)

[54] Charpentier MJE Boulet M Drea CM 2008 Smelling right: the scent of male lemurs advertises genetic quality and relatedness. Mol. Ecol. 17, 3225–3233 (doi:10.1111/j.1365-294X.2008.03831.x) 18565115 10.1111/j.1365-294X.2008.03831.x

[55] Scordato ES Drea CM 2007 Scents and sensibility: information content of olfactory signals in the ringtailed lemur (*Lemur catta*). Anim. Behav. 73, 301–314 (doi:10.1016/j.anbehav.2006.08.006)

[56] Kitchen DM Seyfarth RM Fischer J Cheney DL 2003 Loud calls as indicators of dominance in male baboons (*Papio cynocephalus ursinus*). Behav. Ecol. Sociobiol. 53, 374–384

[57] Clutton-Brock TH Deutsch JC Nefdt RJC 1993 The evolution of ungulate leks. Anim. Behav. 46, 1121–1138 (doi:10.1006/anbe.1993.1302)

[58] Hoglund J Alatalo RV 1995 Leks. Princeton, NJ: Princeton University Press

[59] Clutton-Brock TH Guinness FE Albon SD 1982 Red deer: the behaviour and ecology of two sexes. Chicago, IL: University of Chicago Press

[60] Dubuc C Brent LJN Accamendo AK Gerald MS MacLamon A Semple S Heistermann M Engelhardt A 2009 Sexual skin color contains information about the timing of the fertile phase in free-ranging *Macaca mulatta*. Int. J. Primatol. 30, 777–789 (doi:10.1007/s10764-009-9369-7)

[61] O'Connell SM Cowlishaw G 1994 Infanticide avoidance, sperm competition and mate choice—the function of copulation calls in female baboons. Anim. Behav. 48, 687–694 (doi:10.1006/anbe.1994.1288)

[62] Semple S McComb K Alberts S Altmann J 2002 Information content of female copulation calls in yellow baboons. Am. J. Primatol. 56, 43–56 (doi:10.1002/ajp.1062) 11793412 10.1002/ajp.1062

[63] Berglund A Rosenqvist G 2003 Sex role reversal in pipefish. Adv. Stud. Behav. 32, 131–167 (doi:10.1016/S0065-3454(03)01003-9)

[64] Erckmann WJ 1983 The evolution of polyandry in shorebirds: an evaluation of hypotheses. In Social behaviour of female vertebrates (ed. Wasser SK), pp. 114–168 New York, NY: Academic Press

[65] Amundsen T 2000 Why are female birds ornamented? Trends Ecol. Evol. 15, 149–155 (doi:10.1016/S0169-5347(99)01800-5) 10717684 10.1016/s0169-5347(99)01800-5

[66] Zinner D Nunn C van Schaik CP Kappeler PM 2004 Sexual selection and exaggerated sexual swellings of female primates. In Sexual selection in primates (eds Kappeler PM van Schaik CP), pp. 71–89 Cambridge, UK: Cambridge University Press

[67] Alberts SC Fitzpatrick CL 2012 Paternal care and the evolution of exaggerated sexual swellings in primates. Behav. Ecol. 23, 699–706 (doi:10.1093/beheco/ars052) 10.1093/beheco/ars052 PMC3999376 24771988

[68] Domb LG Pagel M 2001 Sexual swellings advertise female quality in wild baboons. Nature 410, 204–206 (doi:10.1038/35065597) 11242079 10.1038/35065597

[69] Huchard E Courtiol A Benavides JA Knapp LA Raymond M Cowlishaw G 2009 Can fertility signals lead to quality signals? Insights from the evolution of primate sexual swellings. Proc. R. Soc. B 276, 1889–1897 (doi:10.1098/rspb.2008.1923) 10.1098/rspb.2008.1923 PMC2674499 19324772

[70] Plavcan JM 2004 Sexual selection, measures of sexual selection and sexual dimorphism in primates. In Sexual selection in primates (eds Kappeler P Schaik Cv), pp. 230–252 Cambridge, UK: Cambridge University Press

[71] Deschner T Heistermann M Hodges K Boesch C 2003 Timing and probability of ovulation in relation to sex skin swelling in wild West African chimpanzees, *Pan troglodytes verus*. Anim. Behav. 66, 551–560 (doi:10.1006/anbe.2003.2210)

[72] Deschner T Heistermann M Hodges K Boesch C 2004 Female sexual swelling size, timing of ovulation, and male behavior in wild West African chimpanzees. Horm. Behav. 46, 204–215 (doi:10.1016/j.yhbeh.2004.03.013) 15256310 10.1016/j.yhbeh.2004.03.013

[73] Gesquiere LR Wango EO Alberts S Altmann J 2007 Mechanisms of sexual selection: sexual swellings and estrogen concentrations as fertility indicators and cues for male consort decisions in wild baboons. Horm. Behav. 51, 114–125 (doi:10.1016/j.yhbeh.2006.08.010) 17027007 10.1016/j.yhbeh.2006.08.010

[74] Higham JP MacLarnon AM Ross C Heistermann M Semple S 2008 Baboon sexual swelling: information content of size and color. Horm. Behav. 53, 452–462 (doi:10.1016/j.yhbeh.2007.11.019) 18206889 10.1016/j.yhbeh.2007.11.019

[75] Clutton-Brock TH Albon SD Gibson RM Guinness FE 1979 The logical stag: adaptive aspects of fighting in red deer (*Cervus elaphus L*.). Anim. Behav. 27, 211–225 (doi:10.1016/0003-3472(79)90141-6)

[76] Drews C 1996 Contexts and patterns of injuries in free-ranging male baboons (*Papio cynocephalus*). Behaviour 133, 443–474 (doi:10.1163/156853996X00530)

[77] Drews C 1993 The concept and definition of dominance in animal behavior. Behaviour 125, 283–313 (doi:10.1163/156853993X00290)

[78] Hoogland JL 1995 The black-tailed prairie dog: social life of a burrowing mammal. Chicago, IL: University of Chicago Press

[79] Le Boeuf BJ Reiter J 1988 Lifetime reproductive success in northern elephant seals. In Reproductive success (ed. Clutton-Brock TH), pp. 344–362 Chicago, IL: University of Chicago Press

[80] Fernandez-Duque E Huck M 2013 Till death (or an intruder) do us part: intrasexual competition in a monogamous primate. PLoS ONE 8, e53724 (doi:10.1371/journal.pone.0053724) 23372665 10.1371/journal.pone.0053724 PMC3553134

[81] Huchard E Cowlishaw G 2011 Female–female aggression around mating: an extra cost of sociality in a multimale primate society. Behav. Ecol. 22, 1003–1011 (doi:10.1093/beheco/arr083)

[82] Jolly A Pride RE 1999 Troop histories and range inertia of *Lemur catta* at Berenty, Madagascar: a 33-year perspective. Int. J. Primatol. 20, 359–373 (doi:10.1023/A:1020548620372)

[83] McCormick HA MacNulty DR Bosacker AL Lehmane C Bailey A Collins DA Packer C 2011 Male and female aggression: lessons from sex, age, and injury in olive baboons. Behav. Ecol. 23, 684–691 (doi:10.1093/beheco/ars021)

[84] Packer C Pusey AE 1982 Cooperation and competition within coalitions of male lions: kin selection or game theory? Nature 296, 740–742 (doi:10.1038/296740a0)

[85] Carlson AA Young AJ Russell AF Bennett NC McNeilly AS Clutton-Brock T 2004 Hormonal correlates of dominance in meerkats (*Suricata suricatta*). Horm. Behav. 46, 141–150 (doi:10.1016/j.yhbeh.2004.01.009) 15256303 10.1016/j.yhbeh.2004.01.009

[86] Drea CM 2007 Sex and seasonal differences in aggression and steroid secretion in *Lemur catta*: are socially dominant females hormonally ‘masculinized’? Horm. Behav. 51, 555–567 (doi:10.1016/j.yhbeh.2007.02.006) 17382329 10.1016/j.yhbeh.2007.02.006

[87] von Engelhardt N Kappeler PM Heistermann M 2000 Androgen levels and female social dominance in *Lemur catta*. Proc. R. Soc. Lond. B 267, 1533–1539 (doi:10.1098/rspb.2000.1175) 10.1098/rspb.2000.1175 PMC1690709 11007329

[88] Bro-Jørgensen J 2002 Overt female mate competition and preference for central males in a lekking antelope. Proc. Natl Acad. Sci. USA 99, 9290–9293 (doi:10.1073/pnas.142125899) 12089329 10.1073/pnas.142125899 PMC123133

[89] Karvonen E Rintamaki PT Alatalo RV 2000 Female–female aggression and female mate choice on black grouse leks. Anim. Behav. 59, 981–987 (doi:10.1006/anbe.1999.1379) 10860525 10.1006/anbe.1999.1379

[90] Bateman AJ 1948 Intra-sexual selection in *Drosophila*. Heredity 2, 349–368 (doi:10.1038/hdy.1948.21) 18103134 10.1038/hdy.1948.21

[91] Jolly A 2000 Infant killing, wounding and predation in *Eulemur* and *Lemur*. Int. J. Primatol. 21, 21–40 (doi:10.1023/A:1005467411880)

[92] Clutton-Brock TH Albon SD Harvey PH 1980 Antlers, body-size and breeding group size in the Cervidae. Nature 285, 565–567 (doi:10.1038/285565a0)

[93] Ralls K 1976 Mammals in which females are larger than males. Q. Rev. Biol. 51, 245–276 (doi:10.1086/409310) 785524 10.1086/409310

[94] Plavcan JM van Schaik CP Kappeler P 1995 Competition, coalitions and canine size in primates. J. Hum. Evol. 28, 245–276 (doi:10.1006/jhev.1995.1019)

[95] Rand AL 1954 On the spurs on birds’ wings. Wilson Bull. 66, 127–134

[96] Young AJ Bennett NC 2013 Intra-sexual selection in cooperative mammals and birds: why are females not bigger and better armed? Phil. Trans. R. Soc. B 368, 20130075 (doi:10.1098/rstb.2013.0075) 24167305 10.1098/rstb.2013.0075 PMC3826204

[97] Stankowich T Caro T 2009 Evolution of weaponry in female bovids. Proc. R. Soc. B 276, 4329–4334 (doi:10.1098/rspb.2009.1256) 10.1098/rspb.2009.1256 PMC2817105 19759035

[98] Holekamp KE Swale L 2000 Feisty females and meek males: reproductive strategies in the spotted hyena. In Reproduction in context (eds Wallen K Schneider J), pp. 257–285 Cambridge, MA: MIT Press

[99] Drea CM Weldele ML Forger N Coscia EM Frank LG Licht P Glickman SE 1998 Androgens and masculinisation of genitalia in the spotted hyena *Crocuta crocuta*: effects of prenatal anti-androgens. J. Reprod. Fert. 113, 117–127 (doi:10.1530/jrf.0.1130117) 10.1530/jrf.0.1130117 9713384

[100] Glickman SE Coscia EM Frank LG Licht P Weldele ML Drea CM 1998 Androgens and masculinisation of genitalia in the spotted hyaena (*Crocuta crocuta*). 3. Effects of juvenile gonadectomy. J. Reprod. Fert. 113, 129–135 (doi:10.1530/jrf.0.1130129) 10.1530/jrf.0.1130129 9713385

[101] Licht P Frank LG Pavqi SC Yalcinkaya TM Siiteri PK Glickman SE 1992 Hormonal correlates of ‘masculinization’ in female spotted hyaenas (*Crocuta crocuta*). 2. Maternal and fetal steroids. J. Reprod. Fert. 95, 463–474 (doi:10.1530/jrf.0.0950463) 10.1530/jrf.0.0950463 1518002

[102] Licht P 1998 Androgens and masculinization of genitalia in the spotted hyaena (*Crocuta crocuta*). 1. Urogenital morphology and placental androgen production during fetal life. J. Reprod. Fert. 113, 105–116 (doi:10.1530/jrf.0.1130105) 10.1530/jrf.0.1130105 9713383

[103] Frank LG 1997 Evolution of masculinisation: why do female hyaenas have such a large 'penis’? Trends Ecol. Evol. 12, 58–62 (doi:10.1016/S0169-5347(96)10063-X) 21237973 10.1016/s0169-5347(96)10063-x

[104] Racey PA Skinner JD 1979 Endocrine aspects of sexual mimicry in the spotted hyaena (*Crocuta crocuta*). J. Zool. 187, 315–328 (doi:10.1111/j.1469-7998.1979.tb03372.x)

[105] Hawkins CE Dallaas JF Fowler PA Woodroffe R Racey PA 2002 Transient masculinisation in the Fossa *Cryptoprocta ferox* (Carnivora, Viverridae). Biol. Reprod. 66, 610–615 (doi:10.1095/biolreprod66.3.610) 11870065 10.1095/biolreprod66.3.610

[106] Hofer H East ML 2003 Behavioral processes and costs of co-existence in female spotted hyenas: a life-history perspective. Evol. Ecol. 17, 315–331 (doi:10.1023/A:1027352517231)

[107] Muller MN Wrangham R 2002 Sexual mimicry in hyenas. Q. Rev. Biol. 77, 3–16 (doi:10.1086/339199) 11963460 10.1086/339199

[108] Geist V 1971 Mountain Sheep: a study in behavior and evolution. Wildlife Behavior and Ecology Chicago, IL: University of Chicago Press

[109] Lincoln GA Guinness FE Short RV 1972 The way in which testosterone controls the social and sexual behavior of the red deer stag (*Cervus elaphus*). Horm. Behav. 3, 375–396 (doi:10.1016/0018-506X(72)90027-X)

[110] Lincoln GA 1972 The role of antlers in the behaviour of red deer. J. Exp. Zool. 182, 233–249 (doi:10.1002/jez.1401820208)

[111] DeVore I 1965 Primate behavior: field studies of monkeys and apes. New York, NY: Holt, Rinehart and Winston

[112] Hausfater G 1975 Dominance and reproduction in baboons (*Papio cynocephalus*). A quantitative analysis. Contrib. Primatol. 7, 1–150 1170998

[113] Schaller GB 1963 The Mountain Gorilla: ecology and behavior. Chicago, IL: University of Chicago Press

[114] Goodall J 1968 The behaviour of free-living chimpanzes in the Gombe Stream Reserve. *Anim. Behav. Monogr.* 1, 165–311

[115] Goodall J 1986 The chimpanzees of Gombe. Cambridge, MA: Belknap Press

[116] Alberts SC 2012 Magnitude and sources of variation in male reproductive performances. In The evolution of primate societies (eds Mitani JC Call J Kappeler PM Palombit RA Silk JB). Chicago, IL: University of Chicago Press

[117] Appleby MC 1980 Social rank and food access in red deer stags. Behaviour 74, 294–309 (doi:10.1163/156853980X00519)

[118] Pelletier DL Festa-Bianchet M 2006 Sexual selection and social rank in bighorn rams. Anim. Behav. 71, 649–655 (doi:10.1016/j.anbehav.2005.07.008)

[119] Poole JH 1989 Announcing intent: the aggressive state of musth in African elephants. Anim. Behav. 37, 140–152 (doi:10.1016/0003-3472(89)90014-6)

[120] Poole JH 2011 Longevity, competition and musth: a long-term perspective on male reproductive strategies. In The Amboseli elephants (eds Moss CJ), pp. 272–290 Chicago, IL: University of Chicago Press

[121] Barrette C Vandal D 1986 Social rank, dominance, antler size and access to food in snow-bound wild woodland caribou. Behaviour 97, 118–145 (doi:10.1163/156853986X00342)

[122] Feh C 1999 Alliances and reproductive success in Camargue stallions. Anim. Behav. 57, 705–713 (doi:10.1006/anbe.1998.1009) 10196062 10.1006/anbe.1998.1009

[123] Harcourt AH de Waal FBM 1992 Cooperation and conflict: from ants to anthropoids. In Coalitions and alliances in humans and other animals (eds Harcourt AH Waal FBMd), pp. 493–510 Oxford, UK: Oxford University Press

[124] Schülke O Ostner J 2012 Ecological and social influences on sociality. In The evolution of primate societies (eds Mitani JC Call JC Kappeler PM Palombit RA Silk JB), pp. 195–219 Chicago, IL: University of Chicago Press

[125] Strier KB Chaves PB Mendes SL Fagundes V Di Fiore A 2011 Low paternity skew and the influence of maternal kin in an egalitarian, patrilocal primate. Proc. Natl Acad. Sci. USA 108, 18 915–18 919 (doi:10.1073/pnas.1116737108) 22065786 10.1073/pnas.1116737108 PMC3223441

[126] Schülke O Bhagavatula J Vigilant L Ostner J 2010 Social bonds enhance reproductive success in male macaques. Curr. Biol. 20, 2207–2210 (doi:10.1016/j.cub.2010.10.058) 21093261 10.1016/j.cub.2010.10.058

[127] Altmann SA 1962 A field study of sociobiology of rhesus monkeys, *Macaca mulatta*. Ann. N. Y. Acad. Sci. 102, 338–435 (doi:10.1111/j.1749-6632.1962.tb13650.x) 14012344 10.1111/j.1749-6632.1962.tb13650.x

[128] Cowlishaw G Dunbar RI 1991 Dominance rank and mating success in male primates. Anim. Behav. 41, 1045–1056 (doi:10.1016/S0003-3472(05)80642-6)

[129] Dewsbury DA 1982 Dominance rank, copulatory behavior and differential reproduction. Q. Rev. Biol. 57, 135–159 (doi:10.1086/412672) 7051088 10.1086/412672

[130] Ellis L 1995 Dominance and reproductive success among non-human animals: a cross species comparison. Ethol. Sociobiol. 16, 257–333 (doi:10.1016/0162-3095(95)00050-U)

[131] Dubuc C Muniz L Heistermann M Engelhardt A Widdig A 2011 Testing the priority-of-access model in a seasonally breeding primate species. Behav. Ecol. Sociobiol. 65, 1615–1627 (doi:10.1007/s00265-011-1172-8) 21874084 10.1007/s00265-011-1172-8 PMC3134767

[132] Pereira ME Weiss ML 1991 Female mate choice, male migration and the threat of infanticide in ringtailed lemurs. Behav. Ecol. Sociobiol. 28, 141–152 (doi:10.1007/BF00180991)

[133] Post DG Hausfater G McCuskey SA 1980 Feeding behaviour of yellow baboons (*Papio cynocephalus*): relationship to age, gender and dominance rank. Folia Primatol. 34, 170–195 (doi:10.1159/000155954) 7216001 10.1159/000155954

[134] Archie EA Altmann J Alberts SC 2012 Social status predicts wound healing in wild baboons. Proc. Natl Acad. Sci. USA 109, 9017–9022 (doi:10.1073/pnas.1206391109) 22615389 10.1073/pnas.1206391109 PMC3384186

[135] Sapolsky RM 2004 Social status and health in humans and other animals. Annu. Rev. Anthropol. 33, 393–418 (doi:10.1146/annurev.anthro.33.070203.144000)

[136] Sapolsky RM 2005 The influence of social hierarchy on primate health. Science 308, 648–652 (doi:10.1126/science.1106477) 15860617 10.1126/science.1106477

[137] Sterck EHM Watts DP Van Schaik CP 1997 The evolution of female social relationships in nonhuman primates. Behav. Ecol. Sociobiol. 41, 291–309 (doi:10.1007/s002650050390)

[138] Watts DP 2010 Dominance, power and politics in non-human and human primates. In Mind the gap (eds Kappeler PM Silk JB), pp. 109–138 Berlin, Germany: Springer

[139] Sauther ML Sussman RW Gould L 1999 The socioecology of the ringtailed lemur: thirty-five years of research. Evol. Anthropol. 8, 120–132 (doi:10.1002/(SICI)1520-6505(1999)8:4<120::AID-EVAN3>3.0.CO;2-O)

[140] Archie EA Morrison TA Foley CAH Moss CJ Alberts SC 2006 Dominance rank relationships among wild female African elephants, *Loxodonta africana*. Anim. Behav. 71, 117–127 (doi:10.1016/j.anbehav.2005.03.023)

[141] Cote SD 2000 Dominance hierarchies in female mountain goats: stability, aggressiveness and determinants of rank. Behaviour 137, 1541–1566 (doi:10.1163/156853900502718)

[142] Rutberg AT Greenberg SA 1990 Dominance aggression frequencies and modes of aggressive competition in feral pony mares. Anim. Behav. 40, 322–331 (doi:10.1016/S0003-3472(05)80927-3)

[143] Berman CM 1980 Early agonistic experience and rank acquisition among free-ranging infant rhesus monkeys. Int. J. Primatol. 1, 153–170 (doi:10.1007/BF02735595)

[144] Cheney DL 1977 The acquisition of rank and the development of reciprocal alliances among free-ranging immature baboons. Behav. Ecol. Sociobiol. 2, 303–318 (doi:10.1007/BF00299742)

[145] East ML Höner OP Wachter B Wilhelm K Burke T Hofer H 2009 Maternal effects on offspring social status in spotted hyenas. Behav. Ecol. 20, 478–483 (doi:10.1093/beheco/arp020)

[146] Berman CM Chapais B 2004 Kinship and behavior in primates. Oxford, UK: Oxford University Press

[147] Kapsalis E Berman CM 1996 Models of affiliative relationships among free-ranging rhesus monkeys (*Macaca mulatta*). Behaviour 133, 1209–1234 (doi:10.1163/156853996X00378)

[148] Silk JB 2009 Nepotistic cooperation in non-human primate groups. Phil. Trans. R. Soc. B 364, 3243–3254 (doi:10.1098/rstb.2009.0118) 19805431 10.1098/rstb.2009.0118 PMC2781876

[149] Silk JB 2007 The adaptive value of sociality in mammalian groups. Phil. Trans. R. Soc. B 362, 539–559 (doi:10.1098/rstb.2006.1994) 17363359 10.1098/rstb.2006.1994 PMC2346516

[150] Silk JB 2007 Social components of fitness in primate groups. Science 317, 1347–1349 (doi:10.1126/science.1140734) 17823344 10.1126/science.1140734

[151] Silk JB Beehner JC Bergmann TJ Crockford C Engh AL Moscovice LR Wittig RM Seyfarth RM 2010 Strong and consistent social bonds enhance the longevity of female baboons. Curr. Biol. 20, 1359–1361 (doi:10.1016/j.cub.2010.05.067) 20598541 10.1016/j.cub.2010.05.067

[152] Clutton-Brock TH Albon SD Guinness FE 1982 Competition between female relatives in a matrilocal mammal. Nature 300, 178–180 (doi:10.1038/300178a0)

[153] Pusey A 2012 Magnitude and sources of variance in female reproductive performance. In The evolution of primate societies (eds Mitani JC Call J Kappeler PM Palombit RA Silk JB), pp. 343–366 Chicago, IL: University of Chicago Press

[154] Altmann J Alberts SC 2003 Intraspecific variability in fertility and offspring survival in a non-human primate: behavioral control of ecological and social sources. In Offspring: human fertility behavior in a biodemographic perspective (eds Wachter KW Bulatao RA), pp. 140–169 Washington, DC: National Academy Press

[155] Barton RA Whiten A 1993 Feeding competition among female olive baboons *Papio anubis*. Anim. Behav. 46, 777–789 (doi:10.1006/anbe.1993.1255)

[156] Bulger JB Hamilton WJ 1987 Rank and density correlates of inclusive fitness measures in a natural chacma baboon (*Papio ursinus*) population. Int. J. Primatol. 8, 635–650 (doi:10.1007/BF02735781)

[157] Johnson SE 2003 Life history and the competitive environment: trajectories of growth, maturation, and reproductive output among chacma baboons. Am. J. Phys. Anthropol. 120, 83–98 (doi:10.1002/ajpa.10139) 12489139 10.1002/ajpa.10139

[158] Packer C Collins DA Sindimmo A Goodall J 1995 Reproductive constraints on aggressive competition in female baboons. Nature 373, 60–63 (doi:10.1038/373060a0) 7800039 10.1038/373060a0

[159] Setchell JM Lee PC Wickings EJ Dixson AF 2002 Reproductive parameters and maternal investment in mandrills (*Mandrillus sphinx*). Int. J. Primatol. 23, 51–68 (doi:10.1023/A:1013245707228)

[160] Smuts B Nicolson N 1989 Reproduction in wild female olive baboons. Am. J. Primatol. 19, 229–246 (doi:10.1002/ajp.1350190405) 10.1002/ajp.1350190405 31964006

[161] Wasser SK Norton GW Rhine RJ Klein N Kleindorfer S 1998 Ageing and social rank effects on the reproductive system of free-ranging yellow baboons (*Papio cynocephalus*) at Mikumi National Park, Tanzania. Hum. Reprod. Update 4, 430–438 (doi:10.1093/humupd/4.4.430) 9825857 10.1093/humupd/4.4.430

[162] East ML Hofer H 2010 Social environments, social tactics and their fitness consequences in complex mammalian societies. In Social behaviour (eds Szekely T Moore AJ Komdeur J), pp. 360–390 Cambridge, UK: Cambridge University Press

[163] Holekamp KE Dloniak SM 2009 Maternal effects in fissiped carnivores. In Maternal effects in mammals (eds Maestripieri D Mateo J), pp. 227–255 Chicago, IL: University of Chicago Press

[164] Silk JB 2002 Kin selection in primate groups. Int. J. Primatol. 23, 849–875 (doi:10.1023/A:1015581016205)

[165] Silk JB Alberts SC Altmann J 2003 Social bonds of female baboons enhance infant survival. Science 302, 1331–1334 (doi:10.1126/science.302.5649.1331) 14615543 10.1126/science.1088580

[166] Hausfater G Altmann J Altmann S 1982 Long-term consistency of dominance relations among female baboons (*Papio cynocephalus*). Science 217, 752–755 (doi:10.1126/science.217.4561.752) 17772319 10.1126/science.217.4561.752

[167] Holekamp KE Smale L 1991 Dominance acquisition during mammalian social development—the inheritance of maternal rank. Am. Zool. 31, 306–317

[168] van Noordwijk AJ Van Schaik CP 2004 Sexual selection and the careers of primate males: paternity concentration, dominance acquisition tactics and transfer decisions. In Sexual selection in primates: new and comparative perspectives (eds Kappeler PM Van Schaik CP), pp. 208–284 Cambridge, UK: Cambridge University Press

[169] Dobson FS 1982 Competition for mates and predominant juvenile male dispersal in mammals. Anim. Behav. 30, 1183–1192 (doi:10.1016/S0003-3472(82)80209-1)

[170] Fedigan LM 1993 Sex differences and intersexual relations in adult white-faced capuchins (*Cebus capucinus*). Int. J. Primatol. 14, 853–877 (doi:10.1007/BF02220256)

[171] Huck M Fernandez-Duque E 2012 Children of divorce, effects of adult replacements on previous offspring in Argentinean owl monkeys. Behav. Ecol. Sociobiol. 66, 505–517 (doi:10.1007/s00265-011-1297-9)

[172] Pusey AE 1987 Sex-biased dispersal and inbreeding avoidance in birds and mammals. Trends Ecol. Evol. 2, 295–299 (doi:10.1016/0169-5347(87)90081-4) 21227869 10.1016/0169-5347(87)90081-4

[173] Wolff JO 1993 What is the role of adults in mammalian juvenile dispersal? Oikos 68, 173–176 (doi:10.2307/3545324)

[174] Clutton-Brock T Lukas D 2011 The evolution of social philopatry and dispersal in female mammals. Mol. Ecol. 21, 472–492 (doi:10.1111/j.1365-294X.2011.05232.x) 21883582 10.1111/j.1365-294X.2011.05232.x

[175] Slotow R van Dyk G Poole J Page B Klocke A 2000 Older bull elephants control young males. Nature 408, 425–426 (doi:10.1038/35044191) 11100713 10.1038/35044191

[176] Maggioncalda AM Czekala NM Sapolsky RM 2000 Growth hormone and thyroid stimulating hormone concentrations in captive male orangutans: implications for understanding developmental arrest. Am. J. Primatol. 50, 67–76 (doi:10.1002/(SICI)1098-2345(200001)50:1<67::AID-AJP6>3.0.CO;2-V) 10588436 10.1002/(SICI)1098-2345(200001)50:1<67::AID-AJP6>3.0.CO;2-V

[177] Maggioncalda AM Sapolsky RM Czekala NM 1999 Reproductive hormone profiles in captive male orangutans: implications for understanding developmental arrest. Am. J. Phys. Anthropol. 109, 19–32 (doi:10.1002/(SICI)1096-8644(199905)109:1<19::AID-AJPA3>3.0.CO;2-3) 10342462 10.1002/(SICI)1096-8644(199905)109:1<19::AID-AJPA3>3.0.CO;2-3

[178] Perret M 1992 Environmental and social determinants of sexual function in the male lesser mouse lemur (*Microcebus murinus*). Folia Primatol. 59, 1–25 (doi:10.1159/000156637) 1473776 10.1159/000156637

[179] Young AJ 2009 The causes of physiological suppression in vertebrate societies: a synthesis. In Reproductive success in vertebrates (eds Hager R Jones C), pp. 397–436 Cambridge, UK: Cambridge University Press

[180] Utami SS Goossens B Bruford MW de Ruiter JR van Hooff JA 2002 Male bimatorum and reproductive success in Sumatran orangutans. Behav. Ecol. 13, 643–652 (doi:10.1093/beheco/13.5.643)

[181] Setchell JM Kappeler PM 2003 Selection in relation to the sex in primates. Adv. Stud. Behav. 33, 87–176 (doi:10.1016/S0065-3454(03)33003-7)

[182] Hrdy SB 1974 Male-male competition and infanticide among the langurs (*Presbytis entellus*). Folia Primatol. 22, 19–58 (doi:10.1159/000155616) 4215710 10.1159/000155616

[183] Hrdy SB 1979 Infanticide among animals—review, classification, and examination of the implications for the reproductive strategies of females. Ethol. Sociobiol. 1, 13–40 (doi:10.1016/0162-3095(79)90004-9)

[184] van Schaik CP Janson CH 2000 Infanticide by males and its implications. Cambridge, UK: Cambridge University Press

[185] van Schaik CP 2000 Vulnerability to infanticide by males among mammals: patterns among mammals. In Infanticide by males and its implications (eds van Schaik CP Janson CH), pp. 61–71 Cambridge, UK: Cambridge University Press

[186] van Schaik CP 2000 Infanticide by male primates: the sexual selection hypothesis revisited. In Infanticide by males and its implications (eds Schaik CPv Jansen CH), pp. 27–60 Cambridge, UK: Cambridge University Press

[187] Hiraiwa-Hasegawa M Hasegawa T 1994 Infanticide in non-human primates: sexual selection and local resource competition. In Infanticide and parental care (eds Parmigiani S vom Saal FS), pp. 137–154 Chur, Switzerland: Harwood Academic Publishers

[188] Takahata Y 1985 Adult male chimpanzees kill and eat a male newborn infant: newly observed intragroup infanticide in Mahale Mountain National Park, Tanzania. Folia Primatol. 44, 161–170 (doi:10.1159/000156210)

[189] Arnold W Dittami J 1997 Reproductive suppression in male alpine marmots. Anim. Behav. 53, 53–66 (doi:10.1006/anbe.1996.0277)

[190] Langergraber KE Mitani JC Vigilant L 2007 The limited impact of kinship on cooperation in wild chimpanzees. Proc. Natl Acad. Sci. USA 104, 7786–7790 (doi:10.1073/pnas.0611449104) 17456600 10.1073/pnas.0611449104 PMC1876525

[191] Boesch C Boesch H Vigilant L 2006 Cooperative hunting in chimpanzees: kinship or mutualism? In Cooperation in primates and humans (eds Kappeler PM Van Schaik CP), pp. 139–150 Berlin, Germany: Springer

[192] Clutton-Brock TH Brotherton PNM Smith R McIlrath G Kansky R Gaynor D O'Riain MJ Skinner JD 1998 Infanticide and expulsion of females in a cooperative mammal. Proc. R. Soc. Lond. B 265, 2291–2295 (doi:10.1098/rspb.1998.0573) 10.1098/rspb.1998.0573 PMC1689533 9881475

[193] Wasser SK Barash DP 1983 Reproductive suppression among female mammals: implications for biomedicine and sexual selection theory. Q. Rev. Biol. 58, 513–538 (doi:10.1086/413545) 6686686 10.1086/413545

[194] Huck UW Lisk RD McKay MV 1988 Social dominance and reproductive success in pregnant and lactating golden hamsters (*Mesocricetus auratus*) under seminatural conditions. Physiol. Behav. 44, 313–319 (doi:10.1016/0031-9384(88)90031-5) 3065802 10.1016/0031-9384(88)90031-5

[195] Huck UW Lisk RD Miller KSAB 1988 Progesterone levels and socially-induced implantation failure and fetal resorption in golden hamsters (*Mesocricetus auratus*). Physiol. Behav. 44, 321–326 (doi:10.1016/0031-9384(88)90032-7) 3065803 10.1016/0031-9384(88)90032-7

[196] Lambin X Krebs CJ 1993 Influence of female relatedness on the demography of female Townsend's vole populations in the spring. J. Anim. Ecol. 62, 536–550 (doi:10.2307/5203)

[197] Lambin X Yoccoz NG 1998 The impact of population kin-structure on nestling survival in Townsend's voles *Microtus townsendii*. J. Anim. Ecol. 67, 1–16 (doi:10.1046/j.1365-2656.1998.00181.x)

[198] Clutton-Brock TH Hodge SJ Flower TP Spong GF Young AJ 2010 Adaptive suppression of subordinate reproduction in cooperative mammals. Am. Nat. 176, 664–673 (doi:10.1086/656492) 20846043 10.1086/656492

[199] Digby L 2000 Infanticide by female mammals: implication for the evolution of social systems. In Infanticide by males (eds Schaik CPv Jansen CH), pp. 423–446 Cambridge, UK: Cambridge University Press

[200] Rödel HG Starkloff A Bautista A Friedrich A-C Von Holst D 2008 Infanticide and maternal offspring defence in European rabbits under natural breeding conditions. Ethology 114, 22–31 (doi:10.1111/j.1439-0310.2007.01447.x)

[201] Sackett GP 1981 Receiving severe aggression correlates with foetal gender in pregnant pigtail monkeys. Dev. Psychobiol. 14, 267–272 (doi:10.1002/dev.420140315) 7196358 10.1002/dev.420140315

[202] Clutton-Brock TH 1991 The evolution of parental care. Monographs in Behavior and Ecology Princeton, NJ: Princeton University Press

[203] Silk JB Clark-Wheatley CB Rodman PS Samuels A 1981 Differential reproductive success and facultative adjustment of sex ratios among captive female bonnet macaques (*Macaca radiata*). Anim. Behav. 29, 1106–1120 (doi:10.1016/S0003-3472(81)80063-2)

[204] Emlen ST Demong NJ Emlen DJ 1989 Experimental induction of infanticide in female wattled jacanas. Auk 106, 1–7 (doi:10.2307/4087750)

[205] Stephens ML 1982 Male takeover and possible infanticide by a female northern jacana (*Jacana spinosa*). Anim. Behav. 30, 1253–1254 (doi:10.1016/S0003-3472(82)80219-4)

[206] Lukas D Clutton-Brock TH 2011 Group structure, kinship, inbreeding risk and habitual female dispersal in plural-breeding mammals. J. Evol. Biol. 22, 1337–1343 10.1111/j.1420-9101.2011.02385.x 21955169

[207] Russell AF 2004 Mammals: comparisons and contrasts. In Ecology and evolution of cooperative breeding in birds (eds Koenig W Dickinson J), pp. 210–227 Cambridge, UK: Cambridge University Press

[208] Johnstone RA Cant MA 1999 Reproductive skew and the threat of eviction: a new perspective. Proc. R. Soc. Lond. B 266, 275–279 (doi:10.1098/rspb.1999.0633)

[209] Clutton-Brock T Sheldon BC 2010 The seven ages of pan. Science 237, 1207–1208 (doi:10.1126/science.1187796) 20203037 10.1126/science.1187796

[210] Wrangham RW 1980 An ecological model of female-bonded primate groups. Behaviour 75, 262–300 (doi:10.1163/156853980X00447)

[211] Wahaj SA Place NJ Weldele ML Glickman SE Holekamp KE 2007 Siblicide in the spotted hyena: analysis with ultrasonic examination of wild and captive individuals. Behav. Ecol. 18, 974–984 (doi:10.1093/beheco/arm076)

[212] Barthold J Fichtel C Kappeler P 2009 What is it going to be? Pattern and potential function of natal coat change in sexually dichromatic redfronted lemurs (*Eulemur fulvus rufus*). Am. J. Phys. Anthropol. 138, 1–10 (doi:10.1002/ajpa.20868) 18615575 10.1002/ajpa.20868

[213] Altizer S 2003 Social organization and parasite risk in mammals: integrating theory and empirical studies. Annu. Rev. Ecol. Evol. Syst. 34, 517–547 (doi:10.1146/annurev.ecolsys.34.030102.151725)

[214] Freeland WJ 1976 Pathogens and the evolution of primate sociality. Biotropica 8, 12–24 (doi:10.2307/2387816)

[215] Hill DA Lee PL 1998 Predation risk as an influence on group size in cercopithecoid primates: implications for social structure. J. Zool. 245, 447–456 (doi:10.1111/j.1469-7998.1998.tb00119.x)

[216] Janson CH 1998 Testing the predation hypotheses for vertebrate sociality: prospects and pitfalls. Behaviour 135, 389–410 (doi:10.1163/156853998793066177)

[217] Clutton-Brock TH Parker GA 1992 Potential reproductive rates and the operation of sexual selection. Q. Rev. Biol. 67, 437–456 (doi:10.1086/417793)

[218] Kokko H Klug H Jennions MD 2012 Unifying cornerstones of sexual selection: operational sex ratio, Bateman gradient and the scope for competitive investment. Ecol. Lett. 15, 1340–1351 (doi:10.1111/j.1461-0248.2012.01859.x) 22925080 10.1111/j.1461-0248.2012.01859.x

[219] Clutton-Brock TH Harvey PH 1978 Mammals, resources and reproductive strategies. Nature 273, 191–195 (doi:10.1038/273191a0) 347308 10.1038/273191a0

[220] Andersson M Simmons LW 2006 Sexual selection and mate choice. Trends Ecol. Evol. 21, 296–302 (doi:10.1016/j.tree.2006.03.015) 16769428 10.1016/j.tree.2006.03.015

[221] Cotton S Small J Pomiankowski A 2006 Sexual selection and condition-dependent mate preferences. Curr. Biol. 16, R755–R765 (doi:10.1016/j.cub.2006.08.022) 16950102 10.1016/j.cub.2006.08.022

[222] Kappeler PM 2012 Mate choice. In The evolution of primate societies (eds Mitani JC Call J Kappeler PM Palombit RA Silk JB), pp. 343–366 Chicago, IL: University of Chicago Press

[223] Bonduriansky R 2001 The evolution of male choice in insects: a synthesis of ideas and evidence. Biol. Rev. 76, 305–339 (doi:10.1017/S1464793101005693) 11569787 10.1017/s1464793101005693

[224] Edward DA Chapman T 2011 The evolution and significance of male mate choice. Trends Ecol. Evol. 26, 647–654 (doi:10.1016/j.tree.2011.07.012) 21890230 10.1016/j.tree.2011.07.012

[225] Berglund A Rosenqvist G Bernet P 1997 Ornamentation predicts reproductive success in female pipefish. Behav. Ecol. Sociobiol. 46, 145–150 (doi:10.1007/s002650050327)

[226] Champagne FA 2008 Epigenetic mechanisms and the transgenerational effects of maternal care. Front. Neuroendocrinol. 29, 386–397 (doi:10.1016/j.yfrne.2008.03.003) 18462782 10.1016/j.yfrne.2008.03.003 PMC2682215

[227] Champagne FA Curley JP 2009 The transgenerational influence of maternal care on offspring gene expression and behaviour in rodents. In Maternal effects in mammals (eds Maestripieri D Mateo JM), pp. 182–202 Chicago, IL: University of Chicago Press

[228] Mousseau TA Fox CW 1998 The adaptive significance of maternal effects. Trends Ecol. Evol. 13, 403–407 (doi:10.1016/S0169-5347(98)01472-4) 21238360 10.1016/s0169-5347(98)01472-4

[229] Carranza J 2010 Sexual selection and the evolution of evolutionary theories. Anim. Behav. 79, e5–e6 (doi:10.1016/j.anbehav.2009.08.010)

[230] Clutton-Brock T 2010 We do not need a Sexual Selection 2.0-nor a theory of Genial Selection. Anim. Behav. 79, e7–e10 (doi:10.1016/j.anbehav.2009.10.018)

[231] Roughgarden J 2012 The social selection alternative to sexual selection. Phil. Trans. R. Soc. B 367, 2294–2303 (doi:10.1098/rstb.2011.0282) 22777017 10.1098/rstb.2011.0282 PMC3391423

[232] Roughgarden J Akçay E 2010 Do we need a Sexual Selection 2.0? Anim. Behav. 79, e1–e4 (doi:10.1016/j.anbehav.2009.06.006)

[233] Cumming JM 1994 Sexual selection and the evolution of dance fly mating systems (Diptera: Empididae; Empidinae). Can. Entomol. 126, 907–920 (doi:10.4039/Ent126907-3)

[234] LeBas NR Hockham LR Ritchie MG 2003 Nonlinear and correlational sexual selection on ‘honest’ female ornamentation. Proc. R. Soc. Lond. B 270, 2159–2165 (doi:10.1098/rspb.2003.2482) 10.1098/rspb.2003.2482 PMC1691484 14561280

[235] Moczek AP Emlen DJ 2000 Male horn dimorphism in the scarab beetle, *Onthophagus taurus*: do alternative reproductive tactics favour alternative phenotypes? Anim. Behav. 59, 459–466 (doi:10.1006/anbe.1999.1342) 10675268 10.1006/anbe.1999.1342

[236] Watson NL Simmons LW 2010 Reproductive competition promotes the evolution of female weaponry. Proc. R. Soc. B 277, 2035–2040 (doi:10.1098/rspb.2009.2335) 10.1098/rspb.2009.2335 PMC2880095 20200030

[237] Maynard Smith J 1978 The evolution of sex. Cambridge, UK: Cambridge University Press

[238] Clutton-Brock TH 2004 What is sexual selection? In Sexual selection in primates: new and comparative perspectives (eds Kappeler PM Schaik CPv), pp. 24–36 Cambridge, UK: Cambridge University Press

